# Does a High Amount of Unhydrated Portland Cement Ensure an Effective Autogenous Self-Healing of Mortar?

**DOI:** 10.3390/ma12203298

**Published:** 2019-10-11

**Authors:** Magdalena Rajczakowska, Lennart Nilsson, Karin Habermehl-Cwirzen, Hans Hedlund, Andrzej Cwirzen

**Affiliations:** 1 Department of Civil, Environmental and Natural Resources Engineering, Division of Structural and Fire Engineering, Luleå Tekniska Universitet, 97187 Luleå, Sweden; 2 Skanska, Warfvinges väg 25, 112 74 Stockholm, Sweden

**Keywords:** continued hydration, ultra-high performance concrete, cracking, microstructure, calcite

## Abstract

It is commonly accepted that the autogenous self-healing of concrete is mainly controlled by the hydration of Portland cement and its extent depends on the availability of anhydrous particles. High-performance (HPCs) and ultra-high performance concretes (UHPCs) incorporating very high amounts of cement and having a low water-to-cement ratio reach the hydration degree of only 70–50%. Consequently, the presence of a large amount of unhydrated cement should result in excellent autogenous self-healing. The main aim of this study was to examine whether this commonly accepted hypothesis was correct. The study included tests performed on UHPC and mortars with a low water-to-cement ratio and high cement content. Additionally, aging effects were verified on 12-month-old UHPC samples. Analysis was conducted on the crack surfaces and inside of the cracks. The results strongly indicated that the formation of a dense microstructure and rapidly hydrating, freshly exposed anhydrous cement particles could significantly limit or even hinder the self-healing process. The availability of anhydrous cement appeared not to guarantee development of a highly effective healing process.

## 1. Introduction

Ultra-high performance concrete (UHPC) has received vast attention over the past three decades since its introduction in the 1990s [1,2,3]. This material offers not only a very high compressive strength, exceeding 150 MPa, but also exceptional durability and good workability [3,4,5]. One of the main reasons behind the excellent mechanical properties of the UHPC is the high cement content and a very low water-to-cement ratio (w/c). Consequently, this type of concrete is not an environmentally friendly material due to the high CO_2_ footprint. In Table 1, exemplary calculation of the CO_2_ emissions of UHPC versus ordinary concrete is presented based on the CO_2_ emissions of raw materials found in the literature. In addition, reduction in element thickness due to the higher strength was taken into account. The calculation clearly shows that the total CO_2_ emissions of UHPC is twice as high as ordinary concrete.

Sustainable structural engineering (i.e., reduction of energy and resource consumption as well as CO_2_ emissions during the construction of a structure and its usage) is an urgent matter [8,9]. Concrete, next to steel, is one of the most popular structural materials. Over the years, various modifications to concrete’s composition have been proposed in order to improve its properties, (e.g., [10,11,12,13]). Lowering the carbon footprint of concrete can be achieved, for example, by replacing cement with supplementary cementitious materials [14,15], using recycled aggregate [16,17,18] or enhancing its long-term durability. The former can be done by developing concrete using industrial waste (e.g., fly ash [19] or blast furnace slag [20]), whereas the latter can be achieved by designing a material with self-healing properties.

The autogenous self-healing of concrete is a well-known process. International Union of Laboratories and Experts in Construction Materials, Systems and Structures (RILEM) [21] defines autogenous self-healing as a process which occurs when recovery of a material after sustaining damage involves only its original components. In general, causes of autogenous self-healing can be divided into three main groups: physical, mechanical, and chemical. Physical causes consist of the swelling of the cement matrix; mechanical causes include filling of the cracks with fine particles originating from the broken surface of the concrete or transported via water inside the crack; and the most often cited in the literature chemical causes are related to the continued hydration of cement particles and formation of calcium carbonate [21]. Over the past two decades, extensive research has focused on the autogenous self-healing of concrete aimed at understanding the mechanism and possible efficiency of this process (e.g., [22,23,24]).

Due to high cement content leading to a significant amount of unhydrated cement material potentially available, the self-healing of UHPC is expected to be an efficient process for autogenous self-healing, taking into account the continued hydration mechanism. Few studies focusing on the self-healing of these types of concretes have addressed this topic in recent years, (e.g., [23,25]). This process is affected by various factors, including crack width [23], healing time [25,26,27], temperature [23], addition of crystalline admixture [28], and self-healing exposure type [29]. Cracks below 100 μm have shown good self-healing behavior [23]. A detailed analysis of a self-healing mechanism has not yet been performed; however, calcium silicate hydrate (CSH) as well as calcium carbonate crystals have previously been found as the main self-healing products in cracks [25,28]. The majority of research on this topic has been conducted on 28 day old UHPC specimens and only one study [25,26] proposed a different curing procedure (4 day old sample). The microstructure and chemical composition of the binder matrix develops following the timeline of the hydration processes; thus, the efficiency of the self-healing should also depend on the age of the matrix when cracks are formed. As the cracking of concrete may occur at any time during the life of a structure, it is important to take into account the material’s age. Most of the research on self-healing concrete focuses on cracks which developed at 7 days (e.g., [30]) and 28 days (e.g., [31]). There are only a few studies addressing the self-healing probability of concrete at older ages. For example, the self-healing of engineered cementitious composites (ECCs) was studied 90 days after casting. Engineered cementitious composites are characterized by a low water-to-binder ratio, partial replacement of cement with fly ash, and addition of Polyvinyl alcohol (PVA) fibers. They exhibit ultra-ductile behavior and, therefore, reduced crack width. Engineered cementitious composite specimens were found to have narrower cracks (around 80 µm), which lead to a slightly higher healing efficiency [32]. Higher fly ash content (44%) was found to reduce crack width to 40 µm, thus increasing the efficiency of self-healing in older concrete [33]. Other studies show a similar healing efficiency for 28- and 90- day-old ECCs [34]. Six-month-old ECCs did not exhibit any differences in healing when compared with a younger concrete [35]. Age was found to have a secondary effect on the healing kinetics of ordinary 6-month-old mortars with crack widths up to 200 µm [36]. Older specimens contained a higher amount of Portlandite while younger more unhydrated cement particles. This composition could be the cause for a lack of substantial changes in healing with increasing age of concrete material. The authors of Reference [37] also proposed a similar hypothesis based on the results of their study on 11-month-old high-performance fiber-reinforced cementitious composite. On the other hand, the authors of Reference [38] observed slower healing for 90-day-old medium-early-strength ECC due to the reduced availability of the unhydrated material.

The present study aimed first to verify whether the commonly accepted hypothesis assuming that concrete containing high amounts of unhydrated Portland cement particles has a strong natural capability to autogenously self-heal in humid or wet conditions was true. This prediction was simulated by testing UHPC containing an extremely high amount of Portland cement, large amount of silica fume, and a very low water-to-binder ratio. The second simulated case included high-performance mortars with a moderately high amount of cement and a regular water-to-cement ratio. Second, this study aimed to investigate the effect of a 12-month long aging period on the self-healing process of UHPC mortar, as well as to determine the possible differences between the type of healing products forming on the crack surface and inside the crack.

## 2. Materials and Methods

### 2.1. Materials

Three types of mortar mixes were tested: ultra-high performance (U), normal strength mortars based on CEM I 42.5 N (mortar A), and CEM II/A-V 52.5 N Portland fly ash commercial blend with approximately 20% fly ash content (mortar B). The studied mortar mix compositions and basic mechanical properties of the produced mortars are shown in Table 2. The chemical compositions of the applied cements are listed in Table 3.

**Figure 1:**
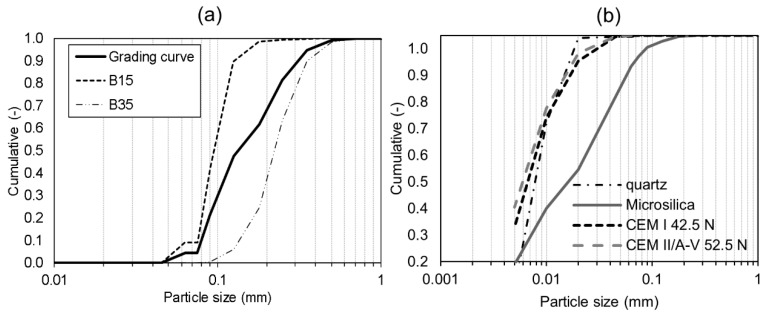
(**a**) Grading curves of the UHPC mix and aggregates; (**b**) grading curves of the quartz, microsilica, and cements applied in the study.

Two types of aggregate (i.e., B15 and B35) provided by Baskarpsand AB with particles smaller than 1 mm were used. The grading curve of each aggregate type as well as the final grading curve of the UHPC mix are presented in Figure 1.

Elkem Microsilica (Oslo, Norway) Grade 920D and Norquartz 45 were used as fillers. BASF (Gothenburg, Sweden) MasterGlenium SKY 600 superplasticizer with polycarboxyl ether polymers was applied in order to achieve the low w/c ratio of the UHPC. In addition, 1.5% vol. of polyvinyl alcohol (PVA) fibers, 5 mm long and with an average thickness of 0.025 mm, were added to each mix. The PVA fibers, due to the fact of their high polarity, were found to boost the self-healing process by supporting the formation of self-healing products, i.e., calcium silicate hydrate (CSH) and Portlandite, acting as the nucleation sites for Ca^2+^ ions [39,40,41]. They have also been proven to limit crack width, in particular in ECCs [33], which increases the efficiency of the autogenous self-healing [35].

### 2.2. Experimental Setup

The experiment consisted of three main parts: cracking of the specimens, assessment of the self-healing efficiency, and, finally, study of the chemical composition and morphology of the healing products formed inside the crack. The efficiency analysis was performed on 4 × 4 × 16 cm samples (L), whereas the investigation of the mechanical properties and morphological analysis was conducted on 1.2 × 1.2 × 6 cm specimens (S).

The cracking was induced 1 day after casting for all samples (A1, B1, U1) and, in addition, after 12 months for the UHPC specimens (U12). Cracks were generated by loading the samples in 3 point bending with a 0.5 mm/s displacement rate, reaching the opening width of approximately 200 µm for large specimens and 100 µm for the small ones. In the next step, all samples were cured by full immersion in tap water at 20 °C for 21 days. No movement or renewal of water was applied.

#### 2.2.1. Self-Healing Efficiency

The self-healing efficiency of the 4 × 4 × 16 cm mortar beams was determined using pulse transmission time measurement performed with a Pundit Lab instrument with exponential transducers of 54 kHz frequency. The measuring procedure followed BS EN 12504-4:2004 [42]. This non-destructive testing method enables evaluation of structural changes connected to the self-healing of a crack [37]. The transmission time for each specimen was measured before cracking (*t_t,0_*), after cracking (*t_t,1_*), as well as after 14 (*t_t,14_*) and 21 (*t_t,21_*) days of healing. In addition, the transmission time recovery ratio, *R_t_*, was calculated as a measure of the self-healing efficiency evolution with time of healing for each mortar mix. The transmission time recovery ratio, *R_t_*, was determined using Equation (1):

(1) $$R_{t,i}=\frac{t_{t,0}-t_{t,i}}{t_{t,0}}$$

where *i* is the moment in time of healing, *t_t,0_* is the transmission time before cracking, and *t_t,i_* is the transmission time at the moment *i* of healing. A positive value of the transmission time recovery ratio, *R_t_*, indicates a beneficial influence of the healing process with a possible closure of the crack, whereas a negative value indicates no healing.

**Figure 2:**
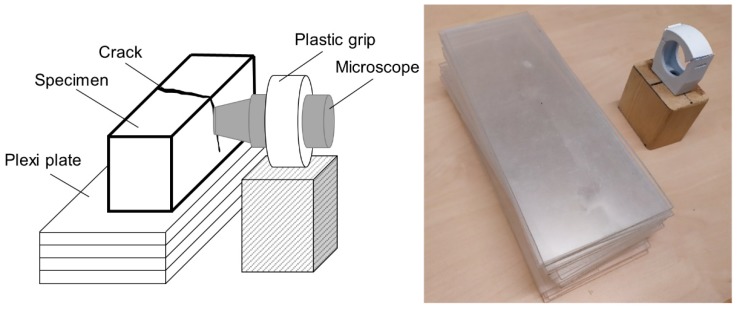
Schematic view of the crack closure testing setup.

Surface crack closure due to the self-healing was evaluated on 4 × 4 × 16 cm mortar specimens using a digital light microscope, type Dino-Lite Pro AM-413T (Dino-Lite Europe, Naarden, The Netherlands) with a 1.3 MP camera and a field of view of 1280 × 1024 pixels. The image spatial resolution was 7 µm per pixel. Images of the crack at the surface of each specimen were acquired after cracking and after 21 days of healing. A special stand was created to control the position of the microscope. In addition, a stand for samples was produced from rectangular 4 mm thick plastic sheet plates. This solution allowed to easily adjust the position of the sample by removing the necessary number of plates (Figure 2).

The images acquired before and after healing were compared and studied using an image processing technique to quantify the healing efficiency. In the first step, the positions of the images in each compared pair were adjusted by applying an image registration method using the Bruker Data Viewer software (Version 1.5.0.2). This enabled an identical position of the crack on the acquired images before and after healing. Subsequently, the images were converted to 8-bit grayscale and filtered with the use of a median filter with a 2-pixel kernel. Cracks formed before and after the healing process were segmented from the image by applying the histogram thresholding method. ImageJ software (Version 1.51) was used for image processing [43]. The computation of the recovery parameters was performed on the binarised images of cracks, i.e., where white pixels (value of 1) depicted the crack area and black pixels (value of 0) the rest of the sample. For each specimen, 4 pairs of images were analyzed, the total analyzed crack length reached approximately 1 cm per image. The global crack closure ratio, *C*, calculated based on the aforementioned images, was defined as in Equation (2):

(2) $$C=\frac{A_{b}-A_{h}}{A_{b}}$$

where *A_b_* and *A_h_* are the area (sum of white pixels) of the crack before and after healing, respectively.

Recovery of the flexural strength was assessed based on testing 1.2 × 1.2 × 6 cm beams. Three samples per set were investigated and the average values were calculated. The mechanical strength recovery index, *S*, was defined as Equation (3):

(3) $$S=\frac{S_{\mathrm{av},h}-S_{\mathrm{av},0}}{S_{\mathrm{av},0}}$$

where *S_av,0_* is the mean value of the flexural strength of the precracked samples. *S_av,h_* depicts the strength of the specimens healed in water for 21 days. A positive value of the strength recovery index, *S*, indicates an increase in strength, whereas a negative value may suggest a lack of effect of the healing process on the mechanical parameters. The first group of specimens was precracked on the same day as the healed samples and stored in the room temperature in the air. Afterwards, they were tested on the same day as the healed (stored in water) samples in order to avoid the effect of the strength increase with the age of the material. This experimental setup was constructed to evaluate how water exposure affected the strength of the specimens with preexisting cracks. 

#### 2.2.2. Microstructure and Healing Products

A Jeol JSM-IT100 scanning electron microscope (SEM) (JEOL Ltd., Tokyo, Japan) with a Bruker energy-dispersive X-ray spectroscope (EDS) (Bruker Corporation, Billerica, MA, USA) was applied for the analysis of the microstructure and healing products. Images were taken in the secondary electron mode (SED) and in the backscattered electron (BSE) mode. Test specimens for each mix were prepared and studied in the following way. After 21 days of healing, samples were dried in the oven for one hour at 45 °C and the crack surface was evaluated for the presence of healing products. In the next step, samples were broken in two halves through the crack and the crack plane was studied. In addition, one extra 12 month old UHPC specimen, U12, was cracked and studied after 1 and 21 days of water exposure. The chemical composition of the healing products and the amount of the unhydrated cement were determined by SEM-EDS analysis. Fragments of specimens A1, U1, and U12 were impregnated in epoxy resin, ground, and polished using Struers CitoVac and Labosystem (Struers, Ballerup, Denmark). A set of grinding plates sprayed with diamond particles having sizes of 9, 3, and 1 µm were used. The amount of the unhydrated cement was calculated based on the BSE image grey histogram by thresholding the white particles [44].

## 3. Results and Discussion

### 3.1. Ultrasound Transmission Time and Flexural Strength Recovery

**Figure 3:**
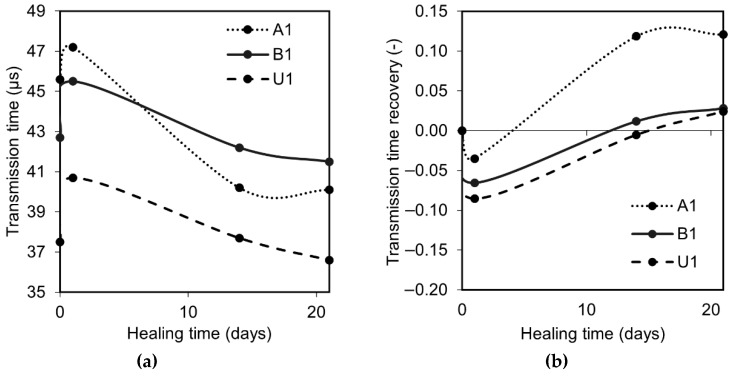
(**a**) Transmission time evolution; (**b**) transmission time recovery ratio evolution.

The transmission time indicated a similar tendency for each of the specimens, i.e., drastic increase after pre-cracking followed by a continuous decrease with time due to the ongoing healing process, Figure 3a. The highest recovery ratio calculated according to Equation (1) was achieved by the sample A1, cracked 1 day after casting. All specimens showed a recovery of the transmission time ratio after approximately 14 days of healing which complied with earlier test results (Figure 3b) [33,38]. Due to the fact that the amount of the unhydrated binder available on the crack plane is very limited, the water-to-cement ratio inside of the crack is significantly higher than in the original cement paste, leading to the acceleration of the cement’s hydration [45]. Therefore, the formation of the healing products is faster at the very beginning. However, later, the process slows down due to the increasingly limited ion transport from the cement matrix and from anhydrous cement particles. The cement particles which are the main source of calcium and silicon ions are covered with a thickening layer of Portlandite, CSH, and presumably also ettringite, thus successively closing up the surface [45].

**Figure 4:**
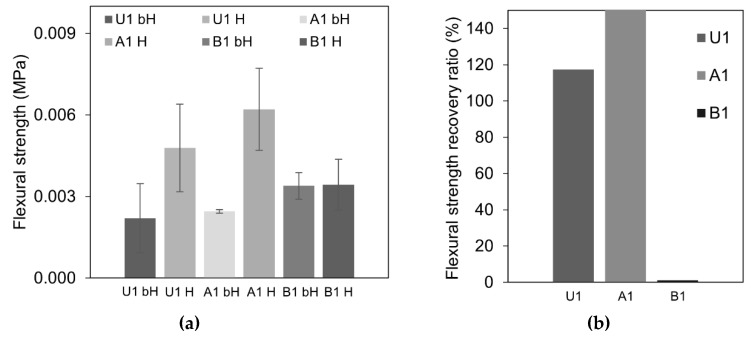
(**a**) Results of the mean flexural strength of cracked samples measured before healing (bH) and after healing (H); (**b**) flexural strength recovery ratio, *S*, for each type of mix.

The calculated flexural strength values for healed and non-healed samples are shown in Figure 4a. The flexural strength recovery ratio, *S*, calculated according to Equation (3) gave an approximate indication of the self-healing effect on the mechanical properties of the cracked samples. All specimens demonstrated a recovery of the flexural strength in comparison with the strength of the pre-cracked samples kept in the air, except for the mix B1 (Figure 4b). The standard deviation indicated a significant variation in the measurements (Figure 4a). This could be related to the different geometrical properties (e.g., length and tortuosity) of the cracked specimens which influenced the failure mechanism.

### 3.2. Crack Closure and Healing Products

**Figure 5:**
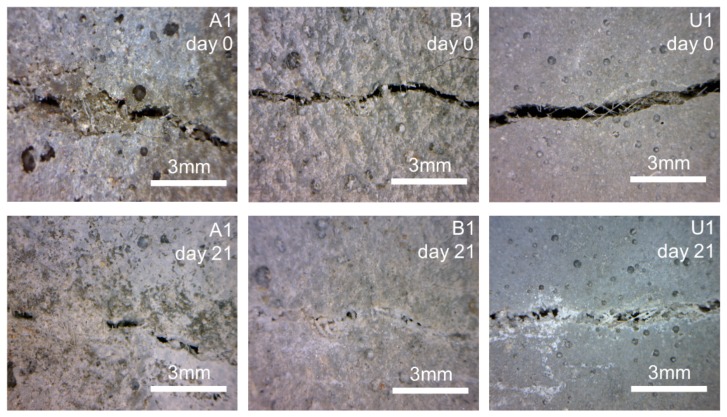
Example of light microscope images of the crack before healing. Four images were taken for each sample: A1; B1; U1 and after 21 days of storage in water: A1; B1; U1.

Light microscope images of representative crack fragments for each specimen obtained directly after cracking and after 21 days of the healing process are shown in Figure 5. The highest visual crack closure was observed in the B1 sample, which is opposite to the observed lack of strength recovery. The obtained crack closure is an external measure of the self-healing efficiency and does not guarantee internal filling of the crack. This might be the justification for the lack of strength recovery in sample B1. White crystalline products were formed on the surface of all specimens as well as on the surface of PVA fibers bridging the cracks (e.g., Figure 5 U1 day 21). The supporting effect of the PVA fibers was also observed earlier and was attributed to the hydrophilic nature of the PVA surface and/or the presence of the OH^−^ groups facilitating the formation of hydration phases [32].

**Figure 6:**
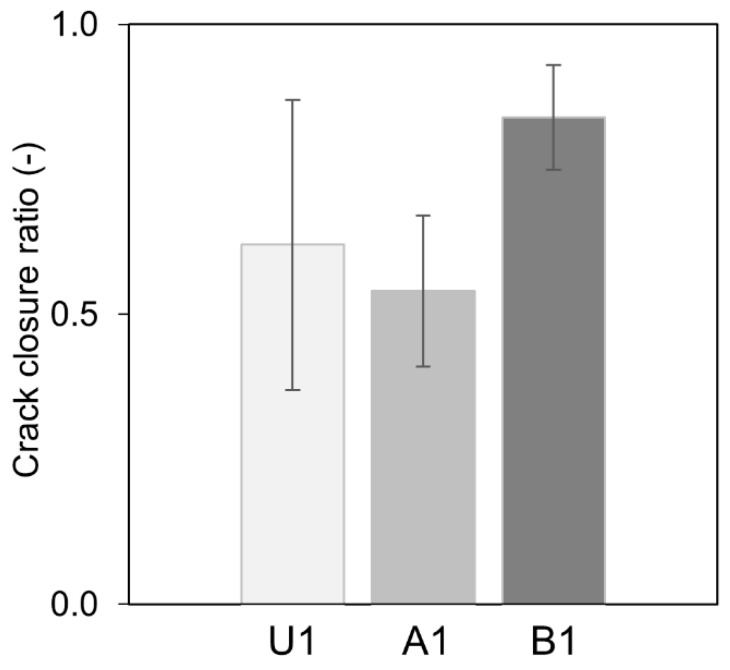
Crack closure ratio for each mortar mix.

The calculated results from the image analysis are listed in Table 4. The quantitative analysis of changes in the detected crack area before and after healing revealed that the most extensive closure was achieved for the sample B1 that contained the fly ash Portland cement (Figure 6). Samples A1 and U1 obtained similar but lower healing efficiency.

**Figure 7:**
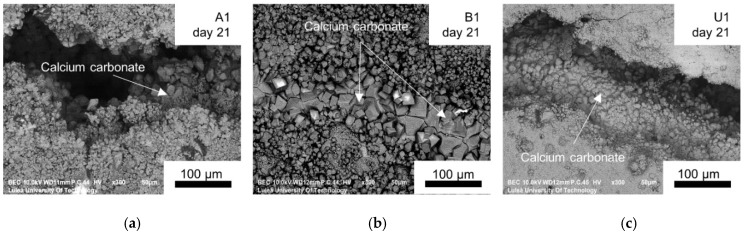
SEM Backscattered Electron (BSE) images (300×) of the crack at the surface for: (**a**) A1; (**b**) B1; (**c**) U1.

Higher resolution SEM study of the healed cracks confirmed the presence of the healing products inside and in the vicinity of the crack edge. The observed cuboid-shaped crystal morphology indicated formation of calcite (Figure 7). In addition, the formation of the calcium carbonate crystals was also observed on the PVA fibers. The presence of other hydration products especially including Portlandite, CSH or ettringite was not detected in any of the studied samples. The formation of those phases cannot be concluded as not occurring inside of the crack, especially in the case of mixes which showed strength recovery after healing (Figure 4). Such analysis was performed on UHPC samples described in the following sections of this publication.

The fly ash blend cement specimens (B1), which did not show regain of flexural strength, had cracks filled mainly with calcium carbonate crystals, indicating the precipitation of calcium carbonate as a dominant process. In this case, the calcium consumed by rapid calcite formation may lead to relatively good external crack closure but small strength recovery, as there is not enough CSH produced inside the crack. This is in contradiction to some of the previous findings [46], where the efficiency of the ongoing hydration mechanism of samples containing fly ash was shown as higher than for samples based on only Portland cement ones. However, studies of self-healing ECCs [35] containing large amounts of fly ash (fly-ash-to-cement-mass ratio>1) revealed primarily calcium carbonate crystals as healing products, suggesting the CaCO_3_ precipitation as the governing mechanism.

### 3.3. Age Effect

**Figure 8:**
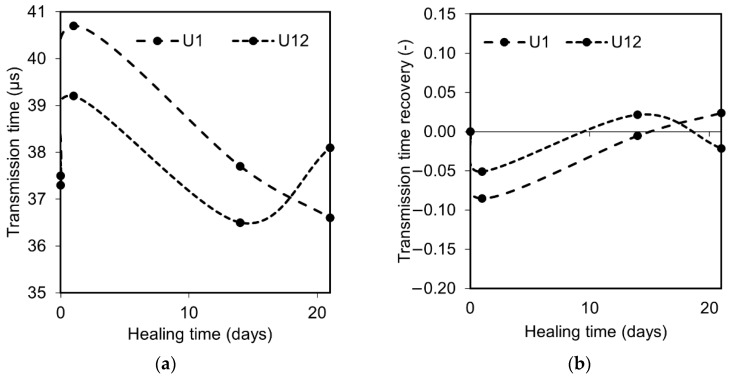
(**a**) Transmission time evolution for samples U1 and U12; (**b**) transmission time recovery ratio evolution for samples U1 and U12.

**Figure 9:**
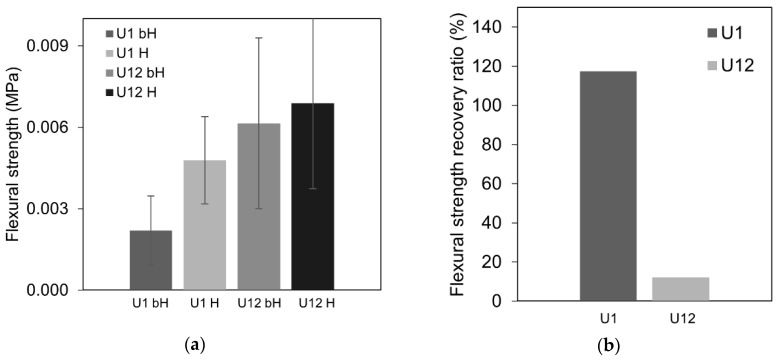
(**a**) Results of the mean flexural strength before healing but after cracking (bH) and after healing of the initially cracked samples (H); (**b**) flexural strength recovery ratio, *S*, calculated according to Equation (3) for mixes U1 and U12.

**Figure 10:**
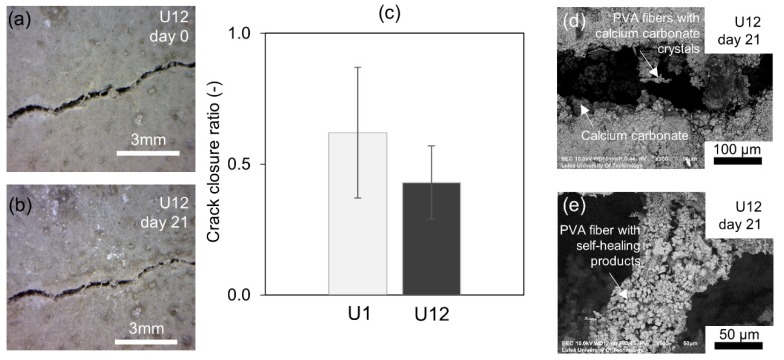
(**a**) Light microscope images of the crack before healing for sample U12; (**b**) light microscope images of the crack after 21 days of healing for sample U12; (**c**) crack closure ratio for samples U1 and U12; (**d**) SEM BSE images (300×) of the crack at the surface for sample U12; (**e**) SEM BSE images (300×) of the self-healing products deposited on the PVA fiber in specimen U12.

Earlier studies indicated that younger concretes should have higher self-sealing efficiency due to the higher amount of anhydrous cement particles present in the solidified binder matrix [45]. On the contrary, the present results showed no clear effect of age on the efficiency and nature of the healing process at least in the case of the studied UHPC concrete. The 12-month-old UHPC (U12) had a similar transmission time recovery rate as the U1 specimen studied at early age (Figure 8a,b). On the other hand, the U1 sample showed a slightly enhanced flexural strength in comparison to the U12, thus indicating formation of different healing products at those two ages (Figure 9a,b). There was no significant age effect present with respect to the surface crack closure ratio (Figure 10c), even though sample U12 showed only a limited amount of healing on the surface of the specimen (Figure 10a,b). The crack closure ratio was comparable for samples U1 and U12.

The SEM evaluation of the surface of the crack again indicated the predominant formation of calcium carbonates as the main healing phase (Figure 10d) with majority deposited on the PVA fibers bridging the crack (Figure 10e).

**Figure 11:**
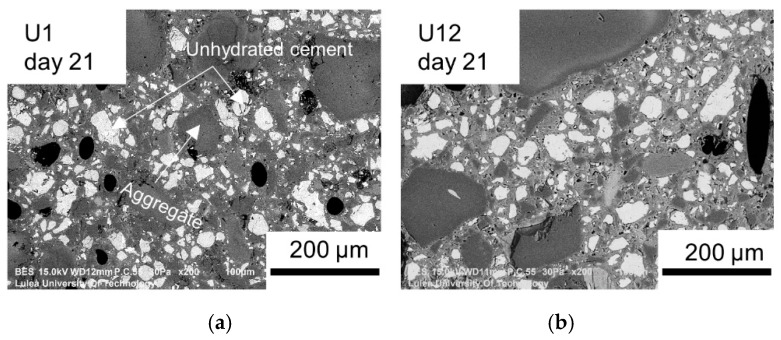
Images of the polished surface of the samples, with white particles indicating unhydrated cement: (**a**) U1; (**b**) U12 (BSE images, 200×).

**Figure 12:**
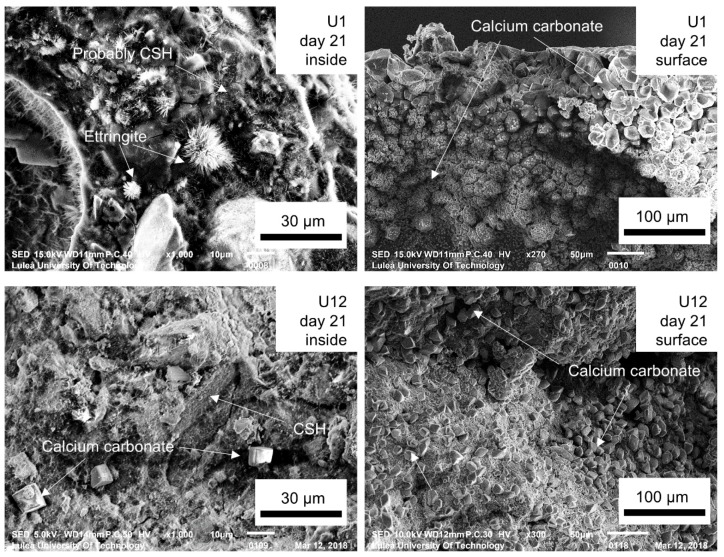
SEM SE images (1000× and ~300×) of the crack plane for U1 and U12.

The calculated crack-filling ratio and the strength recovery are related to two different mechanisms. The crack closure on the surface is connected mainly to the calcite precipitation close to the crack mouth, while the strength recovery is controlled by the formation of the CSH phase. The obtained results could indicate that the CSH formation was less extensive in the aged UHPC samples. To verify that hypothesis, analysis of the resin-impregnated and polished samples was conducted to estimate any potentially significant reduction of the amount of the anhydrous cement due to the aging process (Figure 11). In the next step, the healed samples were split-opened and studied using SEM to identify phases forming inside of the cracks (Figure 12).

The amount of the unhydrated binder was calculated based on SEM BSE images taken 21 days after cracking (Figure 11). The results showed that the amounts of the unhydrated cement in samples U1 and U12 were nearly identical and were equal to 17.02% and 17.34%, respectively. Thus, it did not confirm that the lower strength recovery of the older samples was related to less anhydrous Portland cement present in the binder matrix. Similar results were observed earlier for certain ECC mortars [47]. In that case, the 3-day-old specimens showed more strength recovery than the 90-day-old samples.

A few SEM images of the internal surfaces of the healed cracks are shown in Figure 12. Both non-aged and aged samples revealed the formation of calcite and some also contained CSH. Although not numerically quantified, the analysis clearly indicated that the amount of formed CSH was higher in the non-aged sample. Ettringite only formed in the non-aged samples, which was presumably related to its later transformation to the monosulphate [48]. The filling of cracks with ettringite could possibly contribute to the enhanced flexural strength measured after the healing [49].

**Figure 13:**
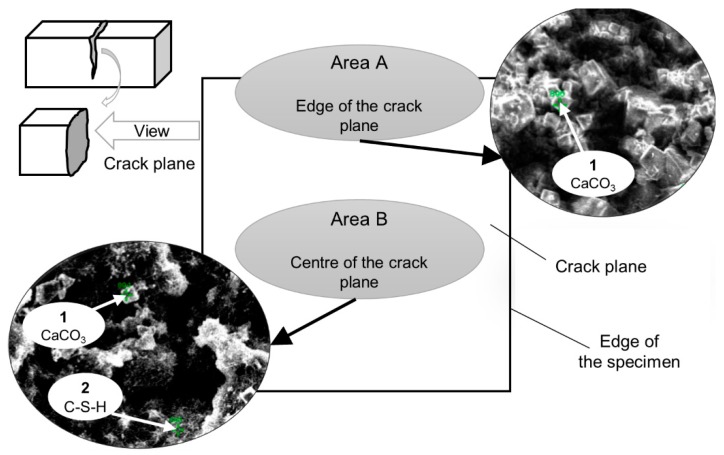
Exemplary spatial distribution of the self-healing products inside the specimens after 21 days (BSE images, 1500×).

The chemical composition of the products was verified by SEM-EDS spot analysis. Two areas of the crack plane were investigated, i.e., close to the edge/crack mouth (Area A, Figure 13) as well as inside of the crack plane (Area B, Figure 13). Two types of products were identified based on the obtained chemical composition (Table 5). Type 1 had a cubic-like morphology, no Si, and was identified as calcite [32,36]. Type 2 had a Ca/Si ratio of approximately 1 [50], a needle-shaped morphology, and was identified as CSH. The needle-shaped products were mainly found inside cracks, whereas the cuboid-like forms, close to the surface (Figure 12).

### 3.4. Autogenous Self-Healing Mechanism—Hypothesis

**Figure 14:**
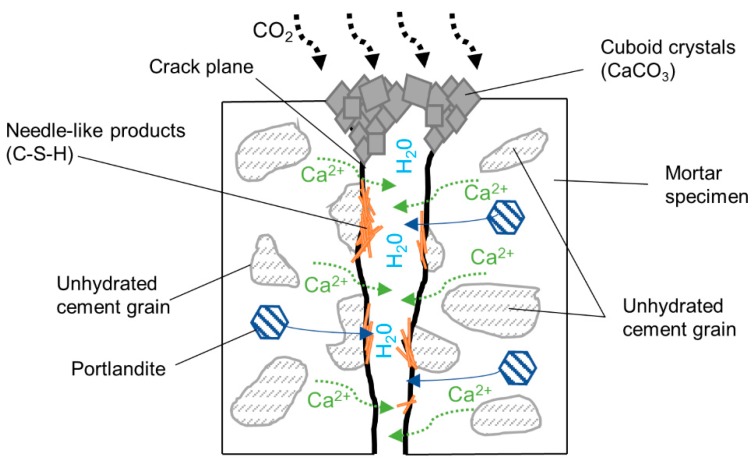
The proposed self-healing mechanism scheme.

Based on a limited number of experimental test results used in this study but combined with the results published by others, a hypothetical mechanism for autogenous self-healing can be outlined. First, it can be assumed that the calcite precipitation mechanism is similar to the carbonation of a Portland cement paste. The CO_2_ from the atmosphere dissolves in water filling the crack and reacts with the calcium ions originating from the cement matrix (Figure 14). The calcium ions originate from the anhydrous cement and Portlandite. The SEM studies of the crack plane indicated that only calcite was formed on the specimen surface and it was the main phase closing the crack entrance. Presumably, the optimum conditions for that process to occur are present only near the crack mouth, thus promoting its preferential formation in that area [51]. The formed cuboid calcite crystals presumably block the opening of the crack and facilitate an increase of ion concentration inside of the crack. Eventually, when supersaturation is reached, other hydration products are formed inside of the closed crack, including CSH, Portlandite, and ettringite [45]. The number of calcium and silicate ions which are transported to the crack is one of the main factors controlling the healing process. Consequently, the permeability of the solidified binder matrix should have a substantial effect on the delivery of required ions from the matrix into the crack. It can be assumed that a denser and less porous matrix will limit or prevent the self-healing process. This assumption was initially confirmed by the results presented in Section 3.3. A significantly lower efficiency of the crack closure was observed in the case of the aged and, thus, denser and less interconnected pores of the UHPC mix U12. This phenomenon may indicate that mortars with more interconnected pores are more capable of self-healing even when containing a smaller amount of the unhydrated binder.

Precipitation of the calcium carbonate at the surface leads to the crack closure, thus contributing to the enhanced durability. On the other hand, the regain in strength could be the measure of the healing mechanism due to the ongoing hydration and formation of the CSH, which results in the increased strength [25,26]. The needle-like product, presumably CSH, was found inside the cracks of the Portland cement-based mortar samples (i.e., A1 and U1). Those samples showed higher strength values.

## 4. Conclusions

The attempt to clarify the effects of the amount of unhydrated cement on the autogenous self-healing process obtained from this preliminary study exposed a number of fundamental questions listed in the formulated conclusions. The main finding clearly indicates a lower self-healing efficiency of the aged UHPC mortar, suggesting that the amount of unhydrated material located in the solidified binder matrix is not the primary factor controlling the process. Instead, the microstructure of the matrix and especially the interconnectivity of the formed pores may be one of the key parameters. The unhydrated cement grains may not provide a sufficient amount of calcium and silicate ions into the solution due to two main phenomena being able to efficiently cut off water access. First, the anhydrous cement may be surrounded by an impermeable water binder matrix. This scenario can occur during ageing when the ongoing hydration densifies the microstructure, common at a very low initial w/c ratio or large amounts of silica fume. Secondly, the hydration reaction developing on the anhydrous cement freshly exposed to water may result in the rapid formation of an impermeable membrane built of ettringite, Portlandite, and CSH. Access to other phases which can provide Ca ions, especially including Portlandite, will be affected as well. Furthermore, the role of secondary cementitious materials can be significant as well, for example, as in the case of the present results where the fly ash promoted closing of the cracks with calcite but also presumably prevented formation of the load bearing CSH. The two outlined potential phenomena should be further investigated to enable the full utilization of the autogenous self-healing process. 

The following conclusions can be formulated based on this initial study and discussion of the hypothetical mechanism of self-healing:

- The OPC mortar (A1) as well as the mortar containing fly ash (B1) pre-cracked at day 1 demonstrated higher self-healing efficiency in comparison with UHPC.
- The presence of fly ash led to the highest crack closure ratio at the surface due to the calcite formation but showed the lowest flexural strength regain indicating limited or a lack of CSH formation.
- The expected higher self-healing efficiency of the mixes containing large amounts of unhydrated Portland cement was not confirmed in this study.
- A low number of interconnected pores limits transport of Ca and Si ions from the solidified matrix to the healed crack, thus hindering the healing process. The densification of the microstructure caused by, for example, ageing can lower or even hinder the autogenous self-healing process.
- Flexural strength recovery is associated with the formation of the CSH and possibly ettringite. The formation of calcite alone does not affect the flexural strength but instead might control conditions in the crack. A more detailed mineralogical analysis of the self-healing products is necessary in order to confirm the results of the chemical composition.

## Figures and Tables

**Table 1:** Environmental impact of ultra-high performance concrete (UHPC) in comparison to ordinary concrete presented in the form of CO_2_ emissions.

Ingredient	CO_2_ Emissions	UHPC	Ordinary Concrete	CO_2_ Emissions	
				UHPC	Ordinary Concrete
	kg/kg	kg/m^3^	kg/m^3^	kg/m^3^	kg/m^3^
Cement	0.931 [6]	1000	400	931	372.4
Water	0.000196 [6]	220	160	0.04312	0.03136
Quartz	0.0234 [7]	300	0	7.02	0
Fine aggregate	0.0026 [6]	700	1100	1.82	2.86
Coarse aggregate	0.0075 [6]	0	730	0	5.475
Silica	1.05 [6]	200	0	210	0
Superplasticizer	0.25 [6]	22.5	0	5.625	0
Element thickness reduction [8]				33%	0
Total CO_2_ emissions (kg/m^3^)				763	381

**Table 2:** Mortar mix compositions.

Ingredient	U kg/m^3^	A kg/m^3^	B kg/m^3^
Cement type	CEM I 42.5 N	CEM I 42.5 N	CEM II/A-V 52.5 N Portland fly ash 20%
w/c	0.22	0.45	0.45
Cement	1000	675	675
Water	220	303.7	303.7
Quartz	300	0	0
B15	350	1196	1196
B35	350	0	0
Condensed Micro silica (Elkem 920)	200	0	0
Superplasticizer	22.5	0	0
PVA fibers	1.5% vol	1.5% vol	1.5% vol
28 day compressive strength (MPa)	143 ± 11	39.5 ± 2.5	36.5 ± 2.0

**Table 3:** Chemical compositions of the used cements.

Chemical Analysis	Mean Value (%)	
	CEM I 42.5 N	CEM II/A-V 52.5 N
CaO	63.30	57.1
SiO_2_	21.20	22.2
Al_2_O_3_	3.40	6.20
Fe_2_O_3_	4.10	3.40
MgO	2.20	2.90
Na_2_O	0.18	0.31
K_2_O	0.56	1.20
SO_3_	2.70	3.50
Cl	<0.01	0.06
Loss of ignition	2.50	-
Water soluble Cr^6+^	<2 mg/kg	<2 mg/kg
Na_2_O-eq.	0.55	1.10

**Table 4:** Results of the crack closure ratio calculation.

Specimen	Area of the Crack before Healing *A_b_* (pixel^2^)	Area of the Crack after 21 Days of Healing *A_h_* (pixel^2^)	Crack Closure Ratio *C*	Mean Value *C_av_*	Standard Deviation
A1	21,624.4	10,637.7	0.51	0.54	0.13
	31,946.1	16,583.8	0.48		
	49,277.3	24,413.4	0.50		
	60,618.8	20,809.5	0.66		
B1	37,418.1	2316.9	0.94	0.84	0.09
	30,833.7	5387.0	0.83		
	31,099.0	7939.7	0.74		
U1	67,726.7	11,798.8	0.83	0.62	0.25
	68,999.1	22,414.1	0.68		
	64,428.9	28,099.9	0.56		
	26,838.6	15,972.5	0.40		

**Table 5:** Results of the EDS measurement.

Elements	Atomic Norm %	
	Product Type 1	Product Type 2
C	18.48	18.45
Ca	19.08	8.39
Si	0.00	7.36
Al	0.00	0.95
O	62.44	63.85
K	0.00	1.00
Ca/Si	0.00	1.14

## References

[1] Azmee N.M., Shafiq N. (2018). Ultra-high performance concrete: From fundamental to applications. Case Stud. Constr. Mater..

[2] Richard P., Cheyrezy M. (1995). Composition of reactive powder concrete. Cem. Concr. Res..

[3] Andrade C., Sanjuán M.A. (1994). Experimental procedure for the calculation of chloride diffusion coefficients in concrete from migration tests. Adv. Cem. Res..

[4] Shi C., Wu Z., Xiao J., Wang D., Huang Z., Fang Z. (2015). A review on ultra high performance concrete: Part I. Raw materials and mixture design. Constr. Build. Mater..

[5] Alkaysi M., El-Tawil S., Liu Z., Hansen W. (2016). Effects of silica powder and cement type on durability of ultra high performance concrete (UHPC). Cem. Concr. Compos..

[6] Kwon S.J., Wang X.Y. (2019). Optimization of the mixture design of low-CO_2_ high-strength concrete containing silica fume. Adv. Civ. Eng..

[7] Lin R.S., Wang X.Y., Zhang G.Y. (2018). Effects of quartz powder on the microstructure and key properties of cement paste. Sustainability.

[8] Randl N., Steiner T., Ofner S., Baumgartner E., Mészöly T. (2014). Development of UHPC mixtures from an ecological point of view. Constr. Build. Mater..

[9] Müller H.S., Haist M., Vogel M. (2014). Assessment of the sustainability potential of concrete and concrete structures considering their environmental impact, performance and lifetime. Constr. Build. Mater..

[10] Berodier E., Gibson II L.R., Burns E., Roberts L., Cheung J. (2019). Robust production of sustainable concrete through the use of admixtures and in-transit concrete management systems. Cem. Concr. Compos..

[11] Duxson P., Provis J.L., Lukey G.C., Van Deventer J.S. (2007). The role of inorganic polymer technology in the development of ‘green concrete’. Cem. Concr. Res..

[12] Gursel A.P., Maryman H., Ostertag C. (2016). A life-cycle approach to environmental, mechanical, and durability properties of “green” concrete mixes with rice husk ash. J. Clean. Prod..

[13] Lima C., Caggiano A., Faella C., Martinelli E., Pepe M., Realfonzo R. (2013). Physical properties and mechanical behaviour of concrete made with recycled aggregates and fly ash. Constr. Build. Mater..

[14] Zhang M.H., Malhotra V.M. (1996). High-performance concrete incorporating rice husk ash as a supplementary cementing material. ACI Mater. J..

[15] Yu J., Lu C., Leung C.K., Li G. (2017). Mechanical properties of green structural concrete with ultrahigh-volume fly ash. Constr. Build. Mater..

[16] Vieira D.R., Calmon J.L., Coelho F.Z. (2016). Life cycle assessment (LCA) applied to the manufacturing of common and ecological concrete: A review. Constr. Build. Mater..

[17] Szeląg M., Zegardło B., Andrzejuk W. (2019). The use of fragmented, worn-out car side windows as an aggregate for cementitious composites. Materials.

[18] Ogrodnik P., Zegardło B., Szeląg M. (2017). The use of heat-resistant concrete made with ceramic sanitary ware waste for a thermal energy storage. Appl. Sci..

[19] Rivera F., Martínez P., Castro J., López M. (2015). Massive volume fly-ash concrete: A more sustainable material with fly ash replacing cement and aggregates. Cem. Concr. Comp..

[20] Shojaei M., Behfarnia K., Mohebi R. (2015). Application of alkali-activated slag concrete in railway sleepers. Mater. Des..

[21] De Rooij M., Van Tittelboom K., De Belie N., Schlangen E. (2013). Self-Healing Phenomena in Cement-Based Materials, State-of-the-Art Report of RILEM Technical Committee 221-SHC..

[22] Jacobsen S., Sellevold E.J. (1996). Self healing of high strength concrete after deterioration by freeze/thaw. Cem. Concr. Res..

[23] Reinhardt H.W., Jooss M. (2003). Permeability and self-healing of cracked concrete as a function of temperature and crack width. Cem. Concr. Res..

[24] Zhong W., Yao W. (2008). Influence of damage degree on self-healing of concrete. Constr. Build. Mater..

[25] Granger S., Pijaudier-Cabot G., Loukili A. Mechanical behavior of self-healed ultra high performance concrete: From experimental evidence to modeling. Proceedings of the 6th international conference on fracture mechanics of concrete and concrete structures.

[26] Granger S., Loukili A., Pijaudier-Cabot G., Chanvillard G. (2007). Experimental characterization of the self-healing of cracks in an ultra high performance cementitious material: Mechanical tests and acoustic emission analysis. Cem. Concr. Res..

[27] Granger S., Cabot G.P., Loukili A., Marlot D., Lenain J.C. (2009). Monitoring of cracking and healing in an ultra high performance cementitious material using the time reversal technique. Cem. Concr. Res..

[28] Escoffres P., Desmettre C., Charron J.P. (2018). Effect of a crystalline admixture on the self-healing capability of high-performance fiber reinforced concretes in service conditions. Constr. Build. Mater..

[29] Kim S., Yoo D.Y., Kim M.J., Banthia N. (2019). Self-Healing capability of ultra-high-performance fiber-reinforced concrete after exposure to cryogenic temperature. Cem. Concr. Comp..

[30] Jiang Z., Li W., Yuan Z. (2015). Influence of mineral additives and environmental conditions on the self-healing capabilities of cementitious materials. Cem. Concr. Comp..

[31] Ahn T.H., Kishi T. (2010). Crack self-healing behavior of cementitious composites incorporating various mineral admixtures. J. Adv. Concr. Technol..

[32] Kan L.L., Shi H.S., Sakulich A.R., Li V.C. (2010). Self-Healing characterization of engineered cementitious composite materials. ACI Mater. J..

[33] Kan L., Shi H. (2012). Investigation of self-healing behavior of engineered cementitious composites (ECC) materials. Constr. Build. Mater..

[34] Alyousif A., Lachemi M., Yildirim G., Şahmaran M. (2015). Effect of self-healing on the different transport properties of cementitious composites. J. Adv. Concr. Technol..

[35] Yang Y., Lepech M.D., Yang E., Li V.C. (2009). Autogenous healing of engineered cementitious composites under wet–dry cycles. Cem. Concr. Res..

[36] Gagné R., Argouges M. (2012). A study of the natural self-healing of mortars using air-flow measurements. Mater. Struct..

[37] Ferrara L., Krelani V., Moretti F., Flores M.R., Ros P.S. (2017). Effects of autogenous healing on the recovery of mechanical performance of high performance fibre reinforced cementitious composites (HPFRCCs): Part 1. Cem. Concr. Comp..

[38] Ma H., Qian S., Zhang Z. (2014). Effect of self-healing on water permeability and mechanical property of medium-early-strength engineered cementitious composites. Constr. Build. Mater..

[39] Nishiwaki T., Sasaki H., Sukmin K. (2015). Experimental study on self-healing effect of FRCC with PVA fibers and additives. J. Ceram. Process. Res..

[40] Choi H., Inoue M., Kwon S., Choi H., Lim M. (2016). Effective crack control of concrete by self-healing of cementitious composites using synthetic fiber. Materials.

[41] Snoeck D. (2015). Self-Healing and Microstructure of Cementitious Materials with Microfibres and Superabsorbent Polymers. Ph.D. Thesis.

[42] British Standards Institution (BSI) (2004). British Standard BS EN 12504-4. Testing Concrete. Determination of Ultrasonic Pulse Velocity.

[43] Schneider C.A., Rasband W.S., Eliceiri K.W. (2012). NIH Image to ImageJ: 25 years of image analysis. Nat. Methods.

[44] Igarashi S.I., Kawamura M., Watanabe A. (2004). Analysis of cement pastes and mortars by a combination of backscatter-based SEM image analysis and calculations based on the powers model. Cem. Concr. Comp..

[45] Huang H., Ye G., Damidot D. (2013). Characterization and quantification of self-healing behaviors of microcracks due to further hydration in cement paste. Cem. Concr. Res..

[46] Van Tittelboom K., Gruyaert E., Rahier H., De Belie N. (2012). Influence of mix composition on the extent of autogenous crack healing by continued hydration or calcium carbonate formation. Constr. Build. Mater..

[47] Yang Y., Yang E.H., Li V.C. (2011). Autogenous healing of engineered cementitious composites at early age. Cem. Concr. Res..

[48] Xu L., Wang P., Zhang G. (2012). Formation of ettringite in Portland cement/calcium aluminate cement/calcium sulfate ternary system hydrates at lower temperatures. Constr. Build. Mater..

[49] Gonzalez M.A., Irassar E.F. (1997). Ettringite formation in low C_3_A Portland cement exposed to sodium sulfate solution. Cem. Concr. Res..

[50] Nonat A. (2004). The structure and stoichiometry of CSH. Cem. Concr. Res..

[51] Sisomphon K., Copuroglu O., Koenders E.A.B. (2012). Self-Healing of surface cracks in mortars with expansive additive and crystalline additive. Cem. Concr. Comp..

